# Design of Fluorescent Probes for Bioorthogonal Labeling of Carbonylation in Live Cells

**DOI:** 10.1038/s41598-020-64790-y

**Published:** 2020-05-06

**Authors:** Hazel Erkan, Dilek Telci, Ozlem Dilek

**Affiliations:** 1 Department of Biotechnology, Yeditepe University, Istanbul, 34755 Turkey; 2 Department of Chemistry, University of Saint Joseph, West Hartford, 06117 Connecticut, USA; 3 Present Address: Gottfried Schatz Research Center for Cell Signaling, Metabolism and Aging, Department of Biophysics, Medical University of Graz, Neue Stiftingtalstrasse, 6/4 8010 Graz, Austria

**Keywords:** Biophysics, Cancer, Chemical biology, Chemistry

## Abstract

With the rapid development of chemical biology, many diagnostic fluorophore-based tools were introduced to specific biomolecules by covalent binding. Bioorthogonal reactions have been widely utilized to manage challenges faced in clinical practice for early diagnosis and treatment of several tumor samples. Herein, we designed a small molecule fluorescent-based biosensor, 2Hydrazine-5nitrophenol (2Hzin5NP), which reacts with the carbonyl moiety of biomolecules through bioorthogonal reaction, therefore can be utilized for the detection of biomolecule carbonylation in various cancer cell lines. Our almost non-fluorescent chemical probe has a fast covalent binding with carbonyl moieties at neutral pH to form a stable fluorescent hydrazone product leading to a spectroscopic alteration in live cells. Microscopic and fluorometric analyses were used to distinguish the exogenous and endogenous ROS induced carbonylation profile in human dermal fibroblasts along with A498 primary site and ACHN metastatic site renal cell carcinoma (RRC) cell lines. Our results showed that carbonylation level that differs in response to exogenous and endogenous stress in healthy and cancer cells can be detected by the newly synthesized bioorthogonal fluorescent probe. Our results provide new insights into the development of novel bioorthogonal probes that can be utilized in site-specific carbonylation labeling to enhance new diagnostic approaches in cancer.

## Introduction

‘Oxidative stress’ is interrelated with an imbalance in the level of reactive oxygen species (ROS) and antioxidants metabolism in the cellular system. Low level of ROS not only induces the activation of transcription factors for oxidative stress adaptation^1^ but also acts as a signaling molecule in cellular processes^2^, which is one of the critical research topics in the field of redox biology. The increased level of ROS and disturbed redox signaling results in the oxidative damage on biomolecules in living systems. Many disorders which can trigger cancer or metabolic diseases are closely correlated with oxidative stress^3,4^.

The dysregulation of ROS levels results in different oxidative modifications in biomolecules such as oxidation, nitration, hydroxylation and carbonylation. Protein carbonylation is the major modification affecting the activity and stability of proteins and this process consists of mainly primary and secondary protein carbonylation mechanisms^5^. Primary protein carbonylation mechanism comprises metal-catalyzed oxidation (MCO) and cleavage of the protein backbone by the α-amidation pathway. Fenton reaction initiates the catalysis of transition in the presence of H_2_O_2_ to produce OH- radicals. Extremely reactive OH^-^ radical leads to direct oxidation of proline, arginine, lysine and threonine^6^. Secondary protein carbonylation is a consequence of covalent adduction either between advanced lipid peroxidation end products (ALEs) or advanced glycation end products (AGEs) with amino acid residues^7^.

Protein carbonylation is chemically irreversible. Decarbonylation process relies on the reduction of carbonyl moiety on proteins via enzymatic processes. Wong and co-workers demonstrated that an inhibitor of thioredoxin reductase can suppress decarbonylation while thioredoxin protein level increases during the decarbonylation process. They claimed that thiol-dependent reduction may support enzymatic decarbonylation processes^8^. In addition, alcohol dehydrogenases, carbonyl reductases and aldo-keto reductases are capable of reducing carbonyl moiety on proteins^9^. Another enzymatic decarbonylation mechanism based on activation of Lon protease, which is activated under stress conditions such as high level of H_2_O_2_ and participates in the degradation of carbonylated proteins^10^. The non-enzymatic endogenous protection of carbonylation is provided by the presence of pyruvate as a free radical scavenger^11^. An increase in the pyruvate concentration relieves the oxidative stress on biomolecules^12^. In addition, serum deprivation induces the generation of ROS^13^, while lack of growth factors may cause apoptotic cell death^14^.

Carbonylation is a highly dynamic post-translational modification. Detection and quantification of carbonylation play a vital role in the determination of the level of oxidative damage^15^ and disease state^16^. Bioorthogonal labeling provides chemo- and regio-selective targeting of the carbonylation in live cells. For example, bioorthogonally prepared probes are easy to monitor real-time imaging and determine the localization and interactome in living cells. 2,4-Dinitrophenyl hydrazine (DNPH) is one of the most common fluorescent probes which is specific for carbonyl moiety on aldehydes and ketones^17^. The usage of DNPH is modified with application in both biochemical and spectrophotometric detection methods of biomolecule carbonylation^18^. Biotin hydrazide probes are designed as DNPH alternatives which permit the application in immunoblotting, spectrophotometric and MS analysis to detect carbonylation of proteins, lipids and glycans^19^. These probes are applicable when DNPH is used for spectrophotometric determination of protein carbonyl content, is that proteins such as cytochrome c and hemoglobin have absorbance wavelengths close enough to DNPH and may interfere with its measurement, leading to inaccurate estimation of protein carbonyls in cellular systems. Recently, hydrazine-tagged coumarin or BODIPY scaffolds are used as fluorescent probes which operate the detection of carbonylation via bioorthogonal labeling in live cells^13,20^. Photostability and solubility of fluorescent probes allow efficiently tracking of a single protein, although cytotoxicity of chemical probes is the major limitation in live cell labeling.

In this study, we synthesized a stable hydrazine-based small molecule, 2-Hydrazine-5-nitrophenol (2Hzin5NP), which reacts with carbonyl groups to produce fluorescent hydrazone, can be utilized for fast monitoring of biomolecule carbonylation in various cancer cell lines using fluorescence methods. Characterization of 2Hzin5NP was confirmed by thin layer chromatography, fluorescence spectroscopy and NMR. The localization of carbonylated proteins was visualized by confocal microscopy. Quantification of protein carbonylation was done by spectrofluorometry. The capacity of 2Hzin5NP to detect differences in protein carbonylation levels due to exogenous and endogenous stress was measured using normal human dermal fibroblasts, A-498 primary site and ACHN metastatic site renal cell carcinoma cell lines as cell models. We demonstrated that 2Hzin5NP can be used in monitoring differential protein carbonylation response in these cells with a distinct molecular background. Our results provide the basis for the development of bioorthogonally designed small molecule that can be used to target site-specific carbonylated groups in cellular environments for further drug delivery and diagnostic systems.

## Results and Discussion

### Synthesis and Structural Characterization of 2-Hydrazine-5-nitrophenol (2Hzin5NP) and its hydrazone products

**Scheme 1:**
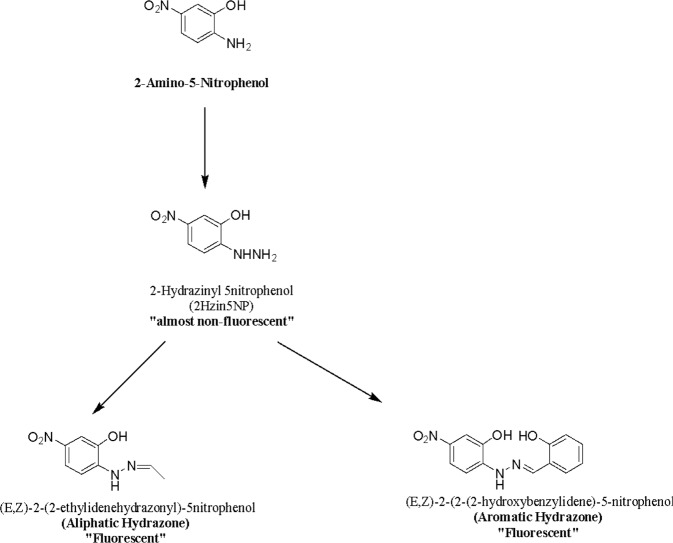
Structure of 2-Hydrazine 5nitrophenol, and its aliphatic hydrazone and aromatic hydrazone.

2Hzin5NP (Scheme 1) was synthesized by diazotization of 2-Amino-5-nitrophenol as described Portoghese *et al*. During organic synthesis, all reactions were monitored by thin layer chromatography (TLC) which provided Rf value for each sample. The product formation and purity were also followed by spectrophotometric and spectrofluorometric analysis. The structural characterization of compounds was determined by NMR, LC-MS and MS-ESI.

In 2Hzin5NP, hydrazine formation was confirmed by the detection of NH peak in 1 H NMR spectrum. While NH protons had a broad peak at 9 ppm, proton of OH was indicated at 10 ppm. Deshielding of NH peak to 10.83 ppm and appearance of CH_3_ peak at 1.31 ppm demonstrated the structural conformation of aliphatic hydrazone with the presence of CH peak at 7.78 ppm. However, impurities and solvent peaks were observed in the ^1^H NMR and ^13^C NMR spectra. Peak of NH_2_ at 4.5 ppm showed the excess hydrazine in the product. Additionally, CH_3_ peak of unreacted acetaldehyde was observed at 2 ppm. Furthermore, solvent peak of ethanol was observed at 1 ppm and presence of water was examined with a sharp peak at 3.5 ppm. The reaction between aromatic aldehyde and 2Hzin5NP resulted in deshielding effect of NH on ^1^H spectrum. Presence of the two OH groups on the aromatic hydrazone led to two similar peaks at 10 ppm and 10.6 ppm. All products were also confirmed by their ^13^C NMR.

To verify the response mechanism of hydrazine probe, 2Hzin5NP towards carbonyl groups, compounds were analyzed by LC-MS. Compound 2Hzin5NP indicated a molecular ion peak of [2Hzin5NP + H]^+^ at m/z = 170.05 in PBS buffer solution. LC-MS spectra of aliphatic and aromatic hydrazone demonstrated that peak of [2Hzin5NP + H]^+^ was absent and new peaks at m/z = 196.07 and at m/z = 274.08 appeared in PBS buffer solution.

**Figure 1:**
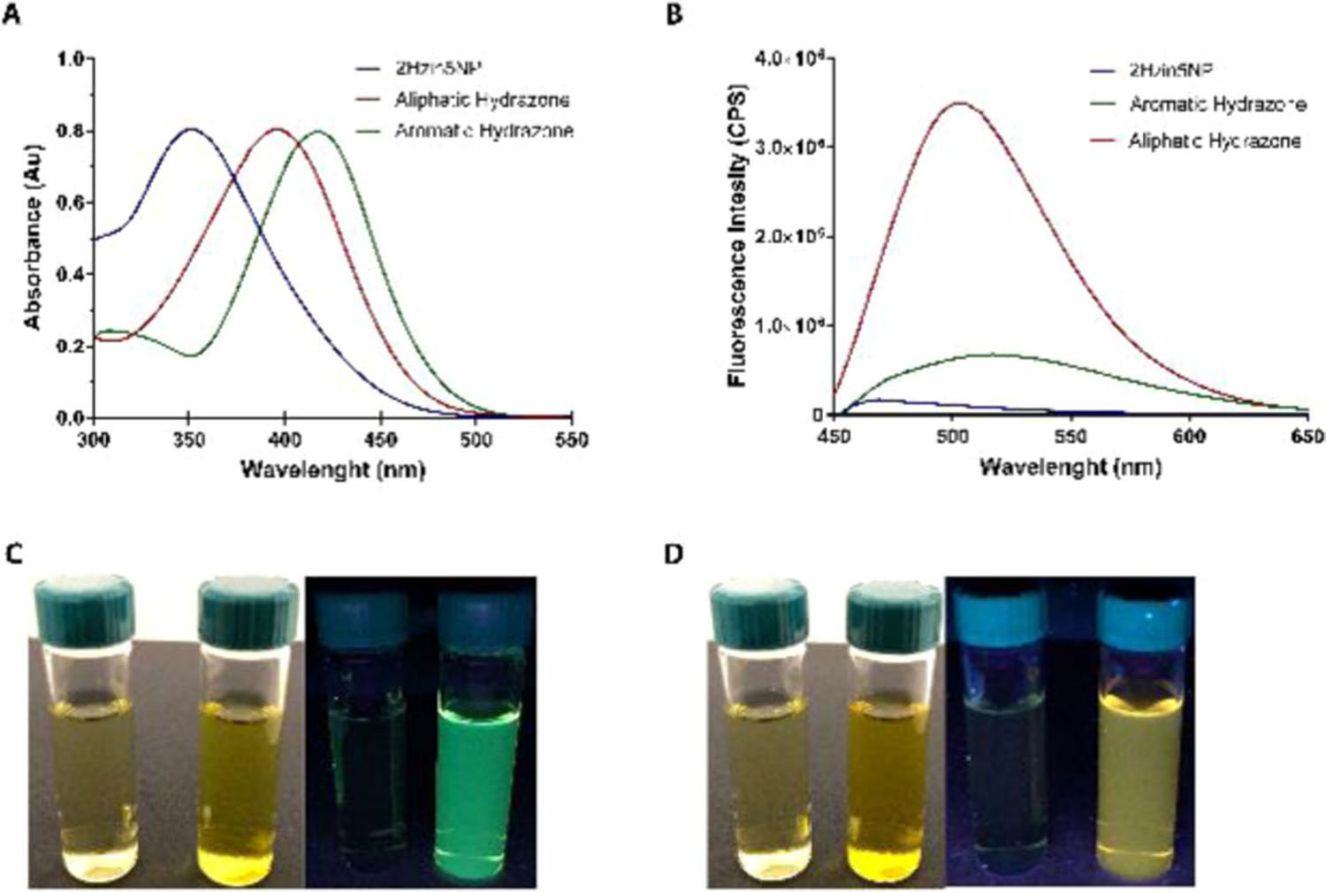
(**A**) Absorption spectra of 2Hzin5NP and its hydrazone products in methanol. From left to right, 2Hzin5NP (blue), aliphatic hydrazone; 2 (red), aromatic hydrazone; 3 (green). (**B**) Emission spectra of compounds in methanol. 2Hzin5NP (blue, λex = 354 nm, λem = 469 nm), 2 (red, λex = 397 nm, λem = 502 nm), 3 (green, λex = 418 nm, λem = 517 nm). (**C**) Solutions of 2Hzin5NP, its aromatic hydrazone in methanol under room light (left) and long wavelength fluorescent light (right). (**D**) Solutions of 2Hzin5NP, its aliphatic hydrazone in methanol under room light (left) and long wavelength fluorescent light (right).

Hydrazine HCl salt is a stable compound in aqueous solutions. We have designed a small molecule-based cell-permeable fluorescent sensor 2Hzin5NP which achieved successful labeling of carbonyls on biomolecules inside live cells. Acetaldehyde and salicylaldehyde were used as mimic molecules of carbonyl groups which correspond to carbonylated biomolecules in cells. The fluorescent probe, 2Hzin5NP, has an absorption maximum at 354 nm and fluorescence emission maximum at 469 nm with the intensity of 2 × 10^5^ CPS. The absorption and emission spectra of 2Hzin5NP (Fig. 1) undergoes a red Stoke shift on reaction with an aliphatic and an aromatic aldehyde on Table 1. While aliphatic hydrazone, provided a 12-fold increase in fluorescence intensity compared to its hydrazine aromatic hydrazone, demonstrated a 3-fold fluorescence intensity increase in MeOH. Fluorescence intensity and color shift changes from hydrazine turn off to hydrazone turn-on transformation were visualized under normal and long wavelength fluorescence light (Fig. 1A). These data also assisted to demonstrate the large difference in fluorescence intensity between an aliphatic and aromatic hydrazone formation. Due to fluorescence quenching of amine derivatives, Hydrazine probe, 2Hzin5NP only showed extremely weak fluorescence as expected at 469 nm (ɸ = 0.001) and addition of aliphatic aldehyde lead to a ‘turn on’ fluorescence emission (ɸ = 0.085) with a bathochromic shift to 502 nm (Fig. 1B, Table 1). Hydrazone formation delocalized the electron distribution on the amide bond increase on the quantum yield of aliphatic and aromatic hydrazone^21^. Since it is highly expected to see a larger emission shift and fluorescence increase on aromatic hydrazone than aliphatic hydrazone due to its conjugation of electron donating electrons through the aromatic ring, cellular systems contain only aliphatic aldehydes.

**Table 1:** Absorption and fluorescence emission spectral data of 2Hzin5NP, and its aliphatic and aromatic hydrazones in methanol.

	Absorption Maximum (nm)	Emission Maximum (nm)	Fluorescence intensity (CPS)	Φ_F_
2Hzin5NP	354	469	170710	0.001
Aliphatic Hydrazone	397	502	3497660	0.085
Aromatic Hydrazone	418	517	680140	0.014

### Bioorthogonal labeling of carbonyls in live cells

Carbonylation of biomolecules is one of the consequences of ROS damage in live cells, which occurs with the addition of aldehyde, ketone or lactam moieties giving rise to the lipid peroxidation, glycation and protein carbonylation. Carbonyl group on biomolecules is also utilized as a chemical reporter for the bioorthogonal labeling. We reasoned that hydrazine-based fluorescent probe can undergo a spectroscopic change upon hydrazone formation as a fluorescent sensor and hence used in the detection of carbonyl functional group on biomolecules in living systems^22,23^. In order to clarify the relative contribution of carbonylation, A-498 primary site and ACHN metastatic site cancer cells along with the normal human dermal fibroblasts, were examined in this study. A-498 cell line is a VHL-mutated while ACHN cell line is a c-MET-overactive cell model of Renal Cell Carcinoma (RCC). VHL protein (pVHL) is responsible for the regulation of Hypoxia-induced factor-1α (HIF-1α) via ubiquitin-mediated degradation by 26 S proteasome^24^. Loss of VHL and increased hypoxia results in overexpression of c-MET^25,26^, which was accompanied by increased metastatic potential and poor prognosis in RCC^27^. Phosphorylated MET activation is suppressed by wild-type VHL gene, hence VHL mutation induces the phosphorylation of MET protein resulting in overactive c-MET pathway. Given that overactive c-MET pathway plays an important role in protection against ROS-induced oxidative stress in renal carcinoma, HDF, A-498 and ACHN cells will possess different systemic levels of oxidative stress hence serve as excellent models to measure the efficiency of 2Hzin5NP probe to monitor relative protein carbonylation levels in a cellular microenvironment.

### Determination of cytotoxic effects of H_2_O_2_ and 2Hzin5NP labeling *in-vitro*

**Figure 2:**
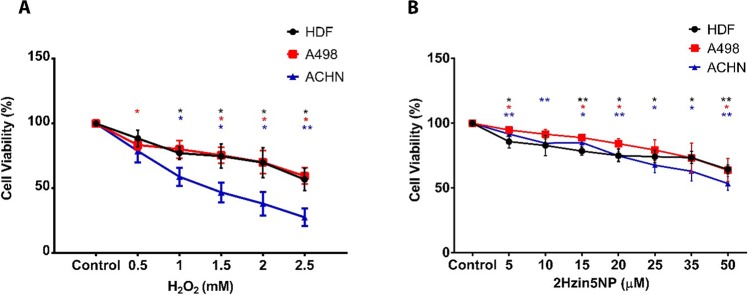
(**A**) Cytotoxic effect of H_2_O_2_ treatment on cell viability of HDF, A498 and ACHN cell lines. Each data point represents the mean percentage of viable cells treated H_2_O_2_ (0.5–2.5 mM) at different time points from three separate experiments. (**B**) Cytotoxic effect of 2Hzin5NP on HDF, A498 and ACHN cell lines. Cells were treated with (5–50 µM) 2Hzin5NP for 30 minutes and then incubated with standard DMEM for 24 hours. Each data point represents the mean percentage of viable cells at different time points from three independent experiments. The percentage of cell viability was calculated by assigning the absorbance value obtained from non-treated cells as 100% for each time point.

The cytotoxicity effects of H_2_O_2_ treatment and 2Hzin5NP labeling were evaluated under *in-vitro* conditions. Cell viability was demonstrated with the colorimetric analysis of formazan formation at 450 nm. H_2_O_2_ treatment of healthy HDF cells with a range of 0.5–2 mM resulted in a maximum of 30% cytotoxicity, while 2.5 mM H_2_O_2_ treatment resulted in a 45% decrease in the cell viability in 24 hours. When A-498 cells were treated with 0.5–1 mM H_2_O_2_, the cell viability was decreased by 20% in 24 hours. Likewise, 1.5–2 mM H_2_O_2_ treatment inhibited the proliferation of A-498 cells within the range of 25–30%. When A-498 cells were incubated with 2.5 mM H_2_O_2_, there was a highly toxic effect seen as a decrease in the cell viability by an average of 40%. The treatment of ACHN cells with 0.5 mM H_2_O_2_ resulted in a 20% decrease in cell viability. Cell viability of 1 mM H_2_O_2_ treated ACHN cells were 60% in 24 hours. When H_2_O_2_ concentration reached to 1.5 mM, cell viability was decreased to 50% for ACHN cell line. 2 mM H_2_O_2_ incubation resulted in a 85% decrease in the ACHN viability, while incubation of ACHN cell line with 2.5 mM H_2_O_2_ caused a significant inhibition of cell proliferation by 70% (Fig. 2A).

Cytotoxicity assay of H_2_O_2_ on HDF, A-498 and ACHN cell lines demonstrated that HDF cells endured higher concentrations of H_2_O_2_, which can be explained by the entrance of HDF into the transition shock state. Shock state is defined as an increase in BCL-2 levels that causes delayed apoptosis to provide enough time for the reversal of cell damage^28^. In addition, as demonstrated by Aryal *et al*., healthy and cancer cells present principal differences in their carbonylation responses to oxidative stress^16^. In redox homeostasis, healthy cells maintain a low level of ROS production and possess antioxidant defenses which are necessary to prevent the oxidative damage. Cancer cells, however are continuously exposed to elevated levels of oxidative stress, which cause the steady-state upregulation of antioxidant defense in order to prevent ROS induced apoptosis^29^. Under oxidative stress conditions, the detoxification process is triggered in order to prevent oxidative damage in healthy cells by neutralizing the high level of ROS, explaining why HDF can endure higher H_2_O_2_ concentrations when compared to the RCC cell lines. On the other hand, A-498 primary site ccRCC cell line could tolerate higher concentrations of H_2_O_2_ compared to the ACHN metastatic site ccRCC cell line. Since A-498 cell line has VHL gene mutation which causes HIF-1α accumulation and activation of several survival pathways including transforming growth factor, epidermal growth factor, insulin-like growth factor and vascular endothelial growth factor, enhanced cell survival against H_2_O_2_ treatment could be possible for A-498 cells^30^.

In our study, HDF, A-498 and ACHN cells were treated with six different concentrations of 2Hzin5NP for 30 minutes to detect the cytotoxic effect of 2Hzin5NP in live cells as shown in Fig. 2B. The labeling of HDF cells with 2Hzin5NP resulted in a 15% decrease of cell viability at the concentrations of 5 µM and 10 µM. Exposure to 2Hzin5NP at 15–35 µM led to a 25% reduction in HDF cell viability, while 50 µM 2Hzin5NP incubation resulted in a 35% decrease in the cell viability. Cytotoxic effect of 5 µM and 10 µM 2Hzin5NP on A-498 cells was recorded as 5 to 10%, while at 15–20 µM 2Hzin5NP, cell viability of A-498 cell line was decreased by a percentage of 15. When A-498 cells were incubated with 25–35 µM 2Hzin5NP, the cell viability was decreased by 20%. Likewise, incubation of A-498 cell line with 2Hzin5NP (50 µM) caused a moderately significant inhibition of cell proliferation by 36%. When ACHN cells were incubated with 5 µM 2Hzin5NP, there was a slightly toxic effect with a decrease in the cell viability by an average of 10%. 10–15 µM 2Hzin5NP incubation resulted in a 15% decrease in the cell viability, while the cytotoxic effect of 20 µM 2Hzin5NP on ACHN cells was recorded as 15%. On the other hand, 2Hzin5NP displayed moderated toxicity with an average of 35% at the concentrations of 25–35 µM in ACHN cell line. Exposure to 2Hzin5NP at 50 µM led to the highest compelling inhibition on ACHN cell proliferation by 45%. In order to do similar effective carbonyl labeling in biomolecules, the maximum dose of 2Hzin5NP within an acceptable toxicity range (up to 20%) was used to label HDF (15 µM), A-498 (20 µM) and ACHN cells (15 µM).

### Fluorescence Labeling of H_2_O_2_-Induced Carbonyls in live cells

**Figure 3:**
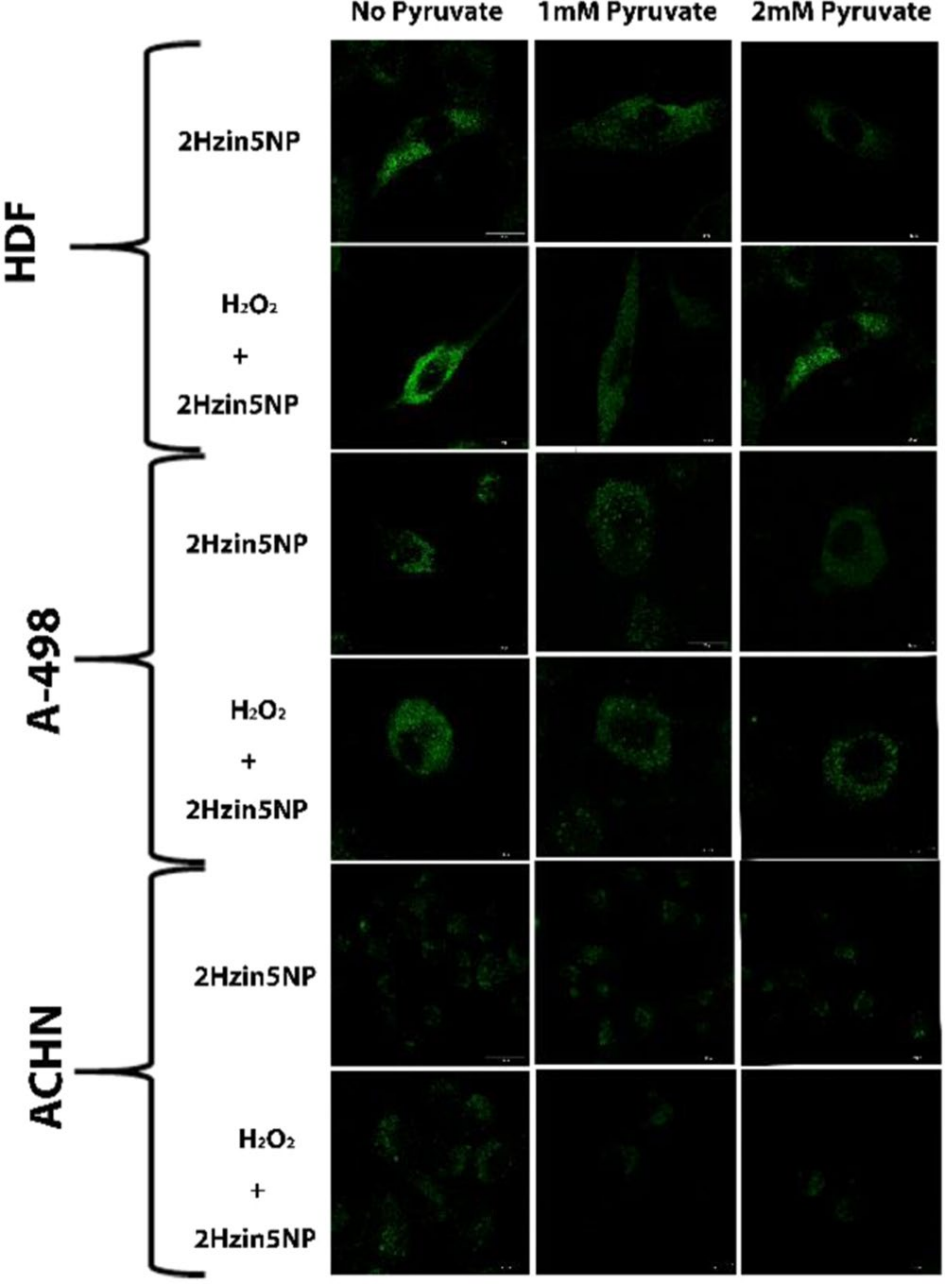
Detection of H_2_O_2_ induced carbonylation levels of HDF, A498 and ACHN cells in the presence and absence of pyruvate. The cells were incubated DMEM with (1 and 2 mM) or without sodium pyruvate prior to 2 mM H_2_O_2_ treatment. Carbonylation was detected by labeling HDF and ACHN cells with 15 µM 2Hzin5NP and A498 with 20 µM 2Hzin5NP for 30 minutes. A 488 nm diode laser was used for excitation and LP 518 filter was used for emission. Images were captured using Zeiss LSM 800 confocal microscope. Figure displays representative images from three independent experiments. Scale bar is equal to 10 µm.

Considering the fluorescence response of 2Hzin5NP towards aldehydes in aqueous solution at physiological pH, we further investigated the labeling efficiency of 2Hzin5NP for H_2_O_2_-induced carbonylation in live cells by confocal microscopy. In addition, we examined the effect of sodium pyruvate in detection of H_2_O_2_-induced carbonylation, since sodium pyruvate is a natural scavenger which reacts with H_2_O_2_ to yield sodium acetate, carbon dioxide and water as byproducts^31^. Physiological concentrations of sodium pyruvate present in cell culture medium also in serum, could alter the effective lifetime of exogenously added H_2_O_2_ and inhibits both oxidative stress damage and H_2_O_2_-induced carbonylation of biomolecules. To verify that, cells were incubated with increased concentrations of sodium pyruvate in FBS containing DMEM and labeled with 2Hzin5NP. As expected, in the absence of pyruvate, cells displayed maximum fluorescence labeling, while increased sodium pyruvate concentration decreased the fluorescence labeling (Fig. 3). Otherwise, detection of the basal level of oxidative stress by 2Hzin5NP labeling demonstrated a visible but faint fluorescent signal for all cells, suggesting that 2Hzin5NP can be used to detect the basal level of protein carbonylation (Fig. 3, top panels). In the absence of pyruvate, H_2_O_2_ treated groups showed dot-like fluorescence staining in cytoplasmic and pre-nuclear regions, indicating the presence of carbonylated protein aggregates. Protein carbonylation is necessary for the recycling of inactivated and misfolded proteins in proteasomal degradation system. Proteasome-dependent degradation of carbonylated proteins depends on the level of carbonylation. While mildly carbonylated proteins can be recognized by proteasomes, highly carbonylated proteins have relatively less ubiquitination site so they cannot be degraded by proteolysis^32^. 20S and 26S proteasomes are responsible for the recognition of carbonyl moieties on proteins and their degradation. While 26S proteasome activity is inhibited by oxidative stress byproducts, the 20 S proteasome is more effective to degrade carbonylated proteins^33^. As a high level of carbonyl moiety on proteins increases hydrophobicity^34^, dysfunctional proteins are accumulated and form protein aggregates called aggresomes^35^, which may induce autophagy^36^ and apoptosis due to their high cytotoxicity in cellular systems^37^. In agreement with this notion, confocal microscopy images suggested the presence of carbonylated protein aggregates that appeared around the nucleus with an even distribution in the cellular cytoplasm (Fig. 3). This result presumably indicates that 2Hzin5NP is membrane-permeable but not nuclear membrane-permeable.

**Figure 4:**
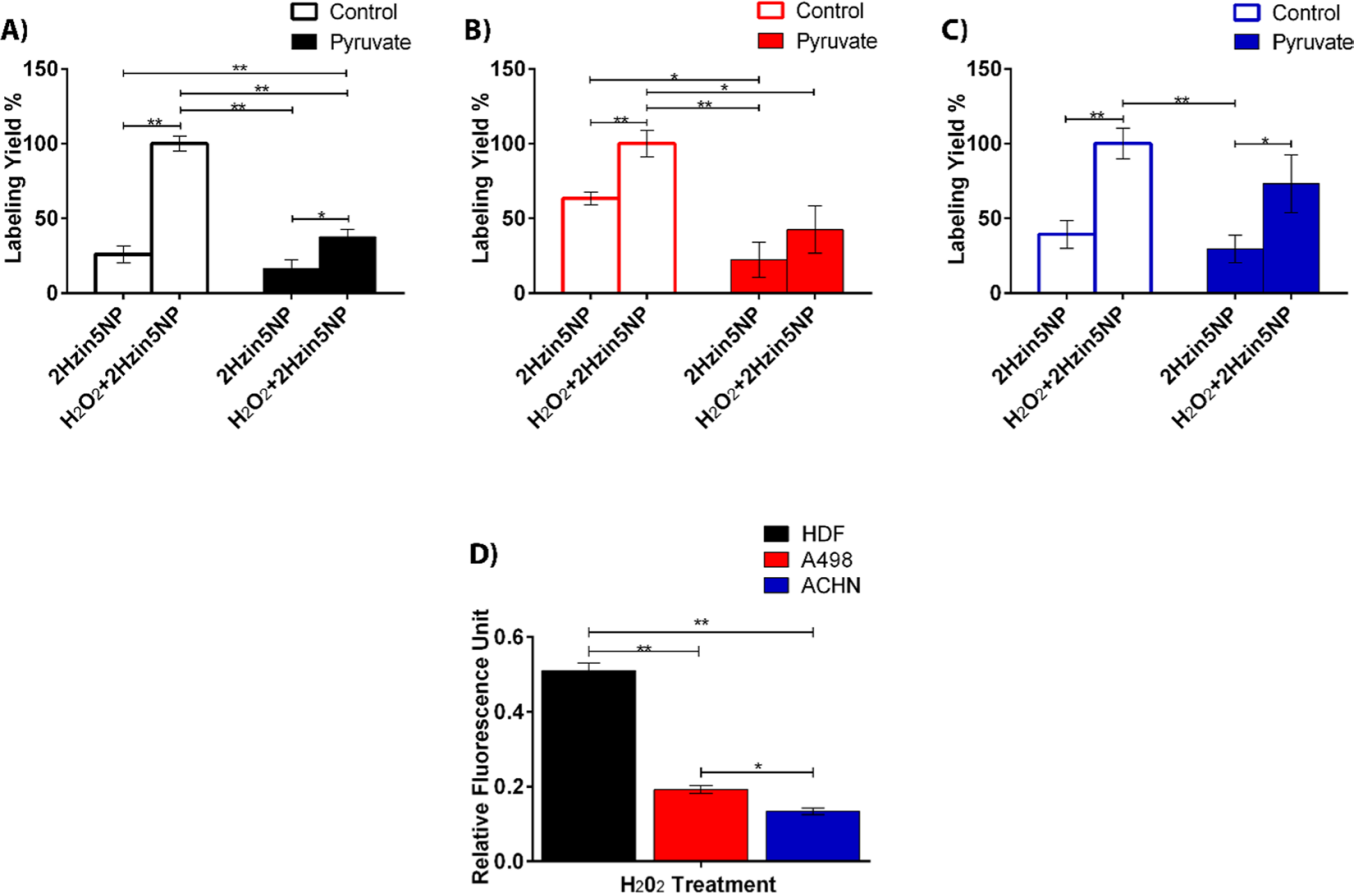
Effect of pyruvate on carbonylation levels in (**A**) HDF, (**B**) A498 and (**C**) ACHN cells. Cells were incubated DMEM with and without 2 mM sodium pyruvate prior to 2 mM H_2_O_2_ treatment. Cells were then lysed by six freeze-thaw cycles and fluorescence intensities were measured by Varioskan Multimode Plate Reader at 396 nm excitation and 506 nm emission. Autofluorescence intensity of control groups was respectively subtracted from all experimental groups. Each data point represents the mean of fluorescence intensity (RFU) at least from three separate experiments. The percentage of RFU obtained for each cell line for H_2_O_2_ + 2Hzin5NP treatment in the absence of pyruvate was set to 100%. (**D**) Detection of carbonylation levels in HDF, A498 and ACHN cells in response to H_2_O_2_ treatment in pyruvate-free conditions. Each data point corresponds to average RFU from three independent experiments. *P ≤ 0.05, **P ≤ 0.01, ***P ≤ 0.001.

Next, we demonstrated the quantitative fluorescence response of 2Hzin5NP towards carbonylated proteins by fluorescence spectrophotometry. A significant increase of the fluorescence intensity at 506 nm was obtained upon following H_2_O_2_ treatment in the absence of sodium pyruvate in all cell lysates. In contrast, a negligible difference in fluorescence intensities was noticed among the treatments in the presence of 2 mM sodium pyruvate in the medium. When HDF cells were incubated with 2 mM pyruvate containing DMEM with the presence of H_2_O_2_, the cells demonstrated 37% fluorescence response as the indication of H_2_O_2_-induced carbonylation level in HDF cell line. While lack of pyruvate led to a 63% increase in fluorescence emission, in HDF cells treated with H_2_O_2_ (Fig. 4A), a 10% difference in fluorescent intensity was detected in the basal redox statue of HDF in the absence of pyruvate. As expected, the fluorescence intensity of 2Hzin5NP labeled cells was decreased by increasing the concentration of pyruvate in the medium. The result is consistent with the findings by Salahudeen *et al*. showing the pyruvate protection of H_2_O_2_ induced damages in renal tissue *in vivo* and *in vitro*^38^. Analysis of carbonylated protein level in A-498 cell line demonstrated that H_2_O_2_ treated A-498 cells generated 42% fluorescent response when cultured in 2 mM pyruvate DMEM (Fig. 4B). There was a 3-fold increase in basal protein-carbonylation levels in response to pyruvate in A-498 cells. 2Hzin5NP labeling of H_2_O_2_ treated ACHN cells exhibited 73% fluorescent labeling in the presence of pyruvate when compared to H_2_O_2_ treated cells grown in the absence of pyruvate, which represents 100% (Fig. 4C). Without H_2_O_2,_ 2Hzin5NP labeled ACHN cells showed 39% fluorescence intensity, which was brought down to 29% with 2 mM pyruvate pre-incubation. When H_2_O_2_-induced carbonylation levels were compared among HDF, A-498 and ACHN cells (Fig. 4D), HDF cells demonstrated 2.6-fold and 3.9-fold higher fluorescent staining than A-498 ACHN, respectively. Comparison of primary site versus metastatic site RCC cell lines showed that A-498 cells had a 1.4-fold higher level of H_2_O_2_ induced carbonylated protein when compared to ACHN. This difference could be due to the VHL mutation in A-498 resulting in HIF1α–induced upregulation of glucose transporters GLUT1-GLUT4^39^ as GLUT-mediated glucose influx stimulates oxidative stress via disruption of cellular energy homeostasis and redox status^40^.

### Fluorescence Labeling of Serum Starvation Induced Carbonyls

**Figure 5:**
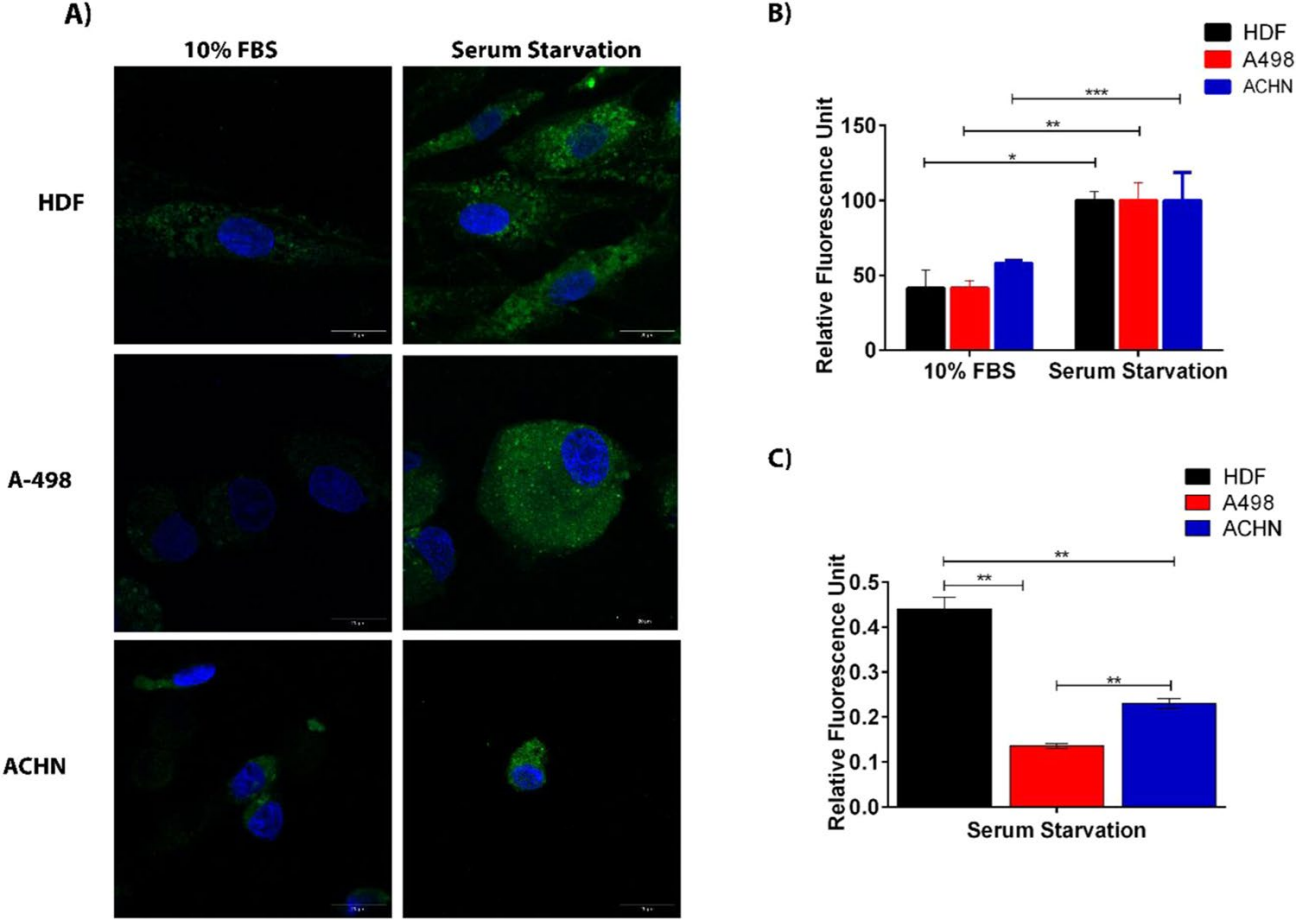
Detection of serum starvation-induced carbonylation in HDF, A498 and ACHN cells. Cells were incubated with and without 10 percent FBS in DMEM for 16 hours. While A498 cells were labeled with 20 µM, ACHN and HDF cells were labeled with 15 µM 2Hzin5NP for 30 minutes. (**A**) Representative images from there independent experiments were captured using Zeiss LSM 800 confocal microscope at 40x objective. 405 nm and 488 nm diode lasers were used for excitation and LP 435 and 518 filters were used for the emission. Scale bar is equal to 10 µm. (**B**) Quantitative analysis of carbonylation levels in HDF, A498 and ACHN cells using Varioskan Multimode Plate Reader. Cell lysates obtained by six freeze-thaw cycles in lysis buffer were analyzed by measuring fluorescence intensity (RFU) at 396 nm excitation and 506 nm emission. The autofluorescence intensity of control groups was respectively subtracted from all experimental groups. Each data point represents average of at least three independent experiments. The percentage of RFU obtained for each 2Hzin5NP labeled cell line in the serum free conditions was set to 100%. (**C**) Detection of carbonylation levels in HDF, A498 and ACHN cells in response to serum starvation. Each data point corresponds to average RFU from three independent experiments. *P ≤ 0.05, **P ≤ 0.01, ***P ≤ 0.001).

Serum starvation is a classical method for endogenous ROS generation, which was introduced by Troppmair and co-workers^41^. Serum starvation causes an alteration in redox homeostasis, which initiates intrinsic cell death pathways^42^. In order to detect the effects of endogenous ROS generation on carbonylation of biomolecules, HDF, A-498 and ACHN cells were serum starved and 2Hzin5NP labeled. In Fig. 5A, confocal images on the right panel, demonstrated an increased carbonylation level with fluorescence staining under the condition of serum deprivation. In 2011, Kuznetsov *et al*. reported that serum starvation caused up to a 3.5 -fold increase in ROS generation in 32D myeloid cells, NIH3T3 mouse embryonic fibroblast cells and in HL-1 cardiac muscle cells^41^. Likewise, in our study, serum starvation led to an increased protein carbonylation levels in healthy cells with respect to cancer cells. In order to verify quantitative results of serum starvation induced carbonylation on live cells, spectrofluorometric analysis was done. RFU obtained for cells that were serum starved and 2Hzin5NP labeled was normalized to 100% labeling yield. When cells were incubated with 10% FBS containing DMEM, fluorescence labeling of HDF and A-498 cell lines was decreased to 41%, while a 58% fluorescence response was detected for ACHN cell line (Fig. 5B). The relative fluorescence level of carbonylated proteins was detected in all cell lines for comparison. Carbonylated protein level of serum starved ACHN cell line was 1.7 times higher than the carbonylation level of serum starved A-498 cell line. While serum starved HDF cells demonstrated a respective 3.6-fold and 1.9-fold higher fluorescent intensity than A-498 and ACHN cells (Fig. 5C) This difference can be explained by the c-MET mutation in ACHN cells or loss of VHL function in A-498 cells^43^ both of which results in direct or indirect activation of the c-MET pathway^44^. Pal and coworkers confirmed that renal tumors overactivate c-MET to cope with ROS-induced oxidative stress^45^. Overexpressed c-Met mediates PI3K/Akt activation which is involved in endogenous ROS generation and oxidative stress^46^. Activation of PI3K/Akt pathway stimulates expression of the redox-sensitive transcription factor nuclear factor-κB (NF-κB), which regulates anti-apoptotic target genes^47^. Therefore, cancer cells evolve to eliminate carbonylated proteins to evade apoptosis. In agreement, we found that A-498 and ACHN cells possess lower levels of carbonylated proteins in response to oxidative stress induced by serum starvation and H_2_O_2_ treatment.

In summary, our results suggest that our novel compound, 2-Hydrazine-5-nitrophenol (2Hzin5NP) is a carbonyl moiety specific bioorthogonal sensor, which is applicable for fluorescent labeling and detection of ROS induced carbonylation in live cells.

## Conclusion

Carbonylation is an irreversible post-translational modification on biomolecules, which can also serve as an oxidative stress biomarker. Determination of carbonylation level of biomolecules may provide information about the distortion of redox biology in cancer cells. Bioorthogonal chemistry provides labeling strategies with the designing of site-specific fluorescent probes to target biomolecules carbonylation via click reaction that gives quantitative and qualitative measurements. Aldehyde - hydrazine reactions are well suited for the detection of carbonylation in live cells. 2-Amino-5-nitrophenol is reduced to 2-Hydrazine-5-nitrophenol by diazotization reaction. 2Hzin5NP is specific for carbonyl groups on aldehydes, ketones and lactams. In this study, 2Hzin5NP was used as a site-specific fluorescent probe for the detection of oxidative stress induced carbonylation in HDF, A498 and ACHN. Primary site and metastatic site of RCC demonstrated different carbonylation levels due to exogenous or endogenous variability in ROS generation.

This site-specific bioorthogonal labeling may be announced as a potentially useful strategy that can be used as a small molecule-based diagnostics for the molecular detection of oxidative damage in biological systems. In future investigations, bioorthogonal *near*-*IR* fluorescent probes would be the next key molecules to synthesize for the detection of carbonylation due to their minimal cellular or tissue components autofluorescence production in the *near*-*IR* region. *Near*-*IR dyes* have the potential to offer highly specific and sensitive fluorescence detection in complex biological systems. It is therefore essential that future probes should have the specificity and selectivity to define oxidative stress induced carbonylation of biomolecules *in vitro* and *in vivo*. Bioorthogonal labeling of carbonylation would allow us to determine the dynamics of oxidative stress induced carbonylation which have vital roles in the diagnosis and determination of therapeutic targets for future cancer therapies.

## Methods

### Materials and Instruments

2-amino-5-nitrophenol, acetaldehyde, salicylaldehyde, tin (II) chloride, phenylmethanesulfonyl fluoride (PMSF), protease inhibitor (PI), hydrogen peroxide (H_2_O_2_) (50%), dimethyl sulfoxide (DMSO), bovine serum albumin (BSA), were purchased from Sigma-Aldrich. Diethyl Ether, Ethanol, Ethyl Acetate, Hexane, Hydrochloric Acid, Methanol, Sodium Chloride, Sodium Nitrite and TLC Aluminum Sheets were obtained from Merck Millipore. Dulbecco’s Modified Eagle’s Medium, High Glucose (DMEM) and Dulbecco’s Modified Eagle’s Medium, High Glucose without pyruvate, Fetal Bovine Serum (FBS) and Penicillin-Streptomycin were purchased from Gibco. Trypsin-EDTA and Dulbecco’s Phosphate Buffered Saline (DPBS) were obtained from Lonza. Primary human kidney epithelial carcinoma (A-498) (Htb-44), metastatic renal cell adenocarcinoma (ACHN) (Crl-1611) and human dermal fibroblast (HDF) (PCS 201-012) cells were purchased from ATCC. Cell proliferation Wst-1 assay, DC protein assay was purchased from Roche, Bio-Rad and Abcam. Solvents and reagents were of analytical grade from commercial suppliers and were used without further purification.

^1^H-NMR and ^13^C-NMR spectra were measured with an AVANCE III 500 MHz spectrometer (Bruker) (TMS as internal standard). The following abbreviations were used to designate chemical shift multiplicities: s = singlet, d = doublet, t = triplet, q = quartet, m = multiplet (denotes complex pattern), dd = doublet of doublets and dt = doublet of triplets. Absorption spectra were obtained by an Agilent/HP 8453 UV-Visible Spectrophotometer using a Starnacell Hellma quartz back wall cuvette with a 1 cm path length. Emission spectra were recorded at room temperature using a Jobin Yvon Horiba FluoroMax-4 spectrofluorometer using a Starnacell Hellma 2 × 10 mm fluorescence cuvette, oriented such that the light passes through the shorter path. In cell culture, live cell imaging was performed by Zeiss Lsm 800 Confocal Microscope and emission spectra of cell lysate was recorded by Thermo Fisher Varioskan Lux Multimode Microplate Reader using a black 96-well microplate.

### Organic synthesis

2Hzin5NP was synthesized adapting reported protocols^48^. Aliphatic Hydrazone was prepared following literature protocol^49^, aromatic hydrazone was synthesized according to the literature procedure^50^.

### Synthesis of 2-Hydrazinyl 5nitrophenol

A cold solution of sodium nitrite (106 mg, 1.5 mmol) in 385 µl water was dropwise added on a cold solution of 2-Amino-5-nitrophenol (200 mg, 1.3 mmol) in 648 µl HCl. The mixture was stirred for 1 hour at −5 °C. Stannous tin chloride (931 mg, 4.1 mmol) was dissolved in 927 µl cold HCl and slowly added on the reaction mixture. The reaction was mixed for 1 hour at −5 °C. The mixture was vacuum filtered, precipitate was washed with cold MeOH and ether. The reaction gave dried 2-Hydrazine-5-nitrophenol HCl salt (177 mg, yield: 66%). ^1^H NMR (500 MHz, DMSO-d6: δ = 10.16 (s, 1 OH), 9.08 (s, 1 NH, broad), 7.75 (dd, 1 H, J = 2.5 Hz, J = 2.5 Hz), 7.59 (d, 1 H, J = 5 Hz), 6.98 (d, 1 H, J = 10 Hz). ^13^C NMR (500 MHz, DMSO-d6): δ = 143.42, 143.13, 138.34, 117.02, 109.88, 108.23. MS-ESI: m/z calculated for C_6_H_7_N_3_O_3_ [M + H]^+^ 170.14, found 170.06.

### Synthesis of (E,Z)-2-(2-ethylidenehydrazonyl)-5-nitrophenol

2Hzin5NP (90 mg, 0.53 mmol) and Acetylaldehyde (29 µl, 0.44 mmol) were stirred in 2.4 ml EtOH for 1 hour at room temperature. The reaction mixture was cooled in an ice bath and the mixture was filtered. Solid product was washed with cold EtOH and dried under vacuum. The reaction gave 90 mg fluorescent product (yield: 87%). ^1^H NMR (500 MHz, DMSO-d6): δ = 10.83 (s, 1 NH), 10.07 (s, 1 OH), 7.78 (d, 1 H, J = 2.5 Hz), 7.09–7.69 (m, 3 H, aromatic), 1.31 (t, 3 H, J = 7 Hz). ^13^C NMR (500 MHz, DMSO-d6): δ = 144.50, 143.47, 141.69, 137.08, 117.66, 115.96, 109.15, 13.73. MS-ESI: m/z calculated for C8H9N3O3 [M + H]^+^ 196.18, found 196.07.

### Synthesis of (E,Z)-2-(2-(2-hydroxybenzylidene)hydrazonyl)-5-nitrophenol

2Hzin5NP (400 mg, 2.36 mmol) and Salicylaldehyde (2.5 ml, 23.6 mmol) were stirred in 19 ml MeOH for 1 hour at room temperature. The reaction mixture was cooled in an ice bath and the mixture was filtered. Solid product was washed with cold MeOH and dried under vacuum. The reaction gave a fluorescent product (155 mg, yield: 24%). The product has Rf: 0.39 value within TLC in Hex:EtOAc (7:4) eluents. ^1^H NMR (500 MHz, DMSO-d6): δ = 10.71 (s, 1 OH), 10.59 (s, 1 NH), 10.25 (s, 1 OH), 8.62 (s, 1 H), 7.79 (d, 1 H, J = 2 Hz), 7.77 (d, 1 H, J = 2 Hz), 7.63 (dd, 1 H, J = 7.5 Hz, J = 2.5 Hz), 7.28 (d, 1 H, J = 9 Hz), 7.21 (t, 2 H, J = 8 Hz), 6.88 (dd, 1 H, J = 8 Hz, J = 7.5 Hz). ^13^C NMR (500 MHz, DMSO-d6): δ = 156.04, 142.18, 141.81, 140.08, 137.91, 130.31, 127.00, 120.49, 119.44, 117.05, 116.05, 109.50, 108.82. MS-ESI: m/z calculated for C_13_H_11_N_3_O_3_ [M + H]^+^ 274.24, found 274.08.

### Fluorescence quantum yield

The fluorescence quantum yields (ΦF) were determined in dilute solutions with an absorbance below 0.1 at the excitation wavelength. Quinine sulfate in 0.1 M H_2_SO_4_ (λex = 347 nm, ΦF = 0.57) was used as a standard^51^. All spectra were recorded with Fluoromax-4 at 23 °C.

Quantum yields were calculated using the following equation:

$${{\Phi }}_{F}^{Sample}={{\Phi }}_{F}^{Standard}\times \frac{({F}^{Sample}-{F}^{Solvent})}{({F}^{Standard}-{F}^{Solvent})}\times \left(\frac{{{\rm{\eta }}}^{Sample}}{{{\rm{\eta }}}^{Standard}}\right)\times \left(\frac{{A}^{Standard}}{{A}^{Sample}}\right)$$

where F denotes the area under the fluorescence band, A denotes the absorbance at the excitation wavelength, and ƞ denotes the refractive index of the solvent. The integration of the emission bands was performed using GraphPad Prism 6.

### Cell culture

HDF, A-498 and ACHN cells were grown in DMEM containing 4.5 g/L glucose, 1 mmol/L sodium pyruvate and 200 mM L-glutamine which was supplemented with 10% (v/v) FBS and 1% (v/v) penicillin-streptomycin in a humidified incubator containing 5% CO_2_ at 37 °C. Once the cells reached to 80% confluent cells, they were sub-cultivated with trypsin–EDTA solution (0.05%).

### WST-1 cell proliferation assay

HDF, A-498 and ACHN cells seeded into 96-well plates overnight were subjected H_2_O_2_ (0.5, 1, 1.5, 2 and 2.5 mM) in FBS free DMEM for 2 h and 2Hzin5NP (5, 10, 15, 20, 25 and 50 µM) in PBS (pH 7.4) for 30 minutes at 37 °C. After the removal of media, cells were washed with PBS and incubated with DMEM at 37 °C for 24 h. The effects of H_2_O_2_ and 2Hzin5NP on cell proliferation were assessed using WST1 assay, which was performed according to the manufacturer’s instructions. Absorbance values were measured at 450 nm and 650 nm by Varioskan Lux Multimode Microplate Reader and percentage of cell viability was calculated by normalizing the values to non-treated control cells, which was adjusted to 100%^52^. All of the measurements were performed three times and the values are presented as the mean ± SD.

### Labeling of carbonylation

HDF, A-498 and ACHN cells were pretreated in complete DMEM with increasing concentrations of (0–2 mM) sodium pyruvate for 1 h at 37 °C. After incubation, cells were treated with 2 mM H_2_O_2_ in FBS free medium at 37 °C for 2 h. After H_2_O_2_ treatment, the medium was discarded and the cells were washed once with PBS. For hydrazine labeling, A-498 cells were incubated with 20 µM 2Hzin5NP while ACHN and HDF cells were subjected to 15 µM 2Hzin5Np in PBS for 30 minutes. To induce endogenous carbonylation cells were serum starved in DMEM up to 24 h and labeled 2Hzin5NP. Cells were imaged using Zeiss LSM 800 confocal microscope at room temperature after cells were washed once with PBS. The samples were excited by a diode 405 nm and 488 lasers and the emission was collected using long pass (LP) 435 and 518 filters.

### Quantification of carbonylation in cell Lysate

Cell pellets of HDF, A-498 and ACHN cells collected were suspended in lysis buffer composed of 0.05 mM PMSF and protease inhibitor cocktail (Santa Cruz Biotechnology Lot: L2315) in dH_2_O. Cells were lysed by freeze-thaw cycles in liquid nitrogen and a water bath at 37 °C. Protein content was determined using DC protein assay according to the manufacturer’s instructions. Standard curve was plotted with BSA standards in the range of 0.05 mg/ml and 1 mg/ml. Fluorescence intensity of H_2_O_2_ treated and labeled cell lysate was measured (Ex: 396 nm, Em: 502 nm) using Varioskan Lux Multimode Microplate Reader.

### Statistical analysis

All data were obtained from three independent experiments and presented as the mean ± SD (error bars). Quantification of carbonylation in cell lysates were analyzed by two-tailed Student t-test. P value less than 0.05 was considered as a statistically significant.

## References

[1] Kiley PJ, Storz G (2004). Exploiting thiol modifications. PLoS biology.

[2] Finkel T (2011). Signal transduction by reactive oxygen species. The Journal of cell biology.

[3] Gill, J. G., Piskounova, E. & Morrison, S. J. Cancer, Oxidative Stress, and Metastasis. Cold Spring Harbor Symposia on Quantitative Biology, 10.1101/sqb.2016.81.030791 (2017). 10.1101/sqb.2016.81.030791 28082378

[4] Muñoz S (2018). Targeting Hepatic Protein Carbonylation and Oxidative Stress Occurring on Diet-Induced Metabolic Diseases through the Supplementation with Fish Oils. Marine drugs.

[5] Wong CM, Bansal G, Marcocci L, Suzuki YJ (2012). Proposed role of primary protein carbonylation in cell signaling. Redox report: communications in free radical research.

[6] Stadtman ER, Berlett BS (1991). Fenton chemistry. Amino acid oxidation. The Journal of biological chemistry.

[7] Jeannette König, T. J., Tilman Grune. Protein Carbonylation in Aging and Senescence. Protein Carbonylation: Principles, Analysis and Biological Implications. (ed. Ros, J.) 272-290 (Wiley, 2017).

[8] Wong CM, Cheema AK, Zhang L, Suzuki YJ (2008). Protein Carbonylation as a Novel Mechanism in Redox Signaling. Circulation Research.

[9] Ellis EM (2007). Reactive carbonyls and oxidative stress: potential for therapeutic intervention. Pharmacology & therapeutics.

[10] Ngo JK, Davies KJA (2009). Mitochondrial Lon Protease is a Human Stress Protein. Free radical biology & medicine.

[11] Shostak A, Gotloib L, Kushnier R, Wajsbrot V (2000). Protective effect of pyruvate upon cultured mesothelial cells exposed to 2 mM hydrogen peroxide. Nephron.

[12] Mukherjee K (2017). Benzocoumarin Hydrazine: A Large Stokes Shift Fluorogenic Sensor for Detecting Carbonyls in Isolated Biomolecules and in Live Cells. ACS Sensors.

[13] Mukherjee K, Chio TI, Sackett DL, Bane SL (2015). Detection of oxidative stress-induced carbonylation in live mammalian cells. Free Radic Biol Med.

[14] Pandey S, Lopez C, Jammu A (2003). Oxidative stress and activation of proteasome protease during serum deprivation-induced apoptosis in rat hepatoma cells; inhibition of cell death by melatonin. Apoptosis: an international journal on programmed cell death.

[15] Gonos ES (2018). Origin and pathophysiology of protein carbonylation, nitration and chlorination in age-related brain diseases and aging. Aging.

[16] Aryal B, Rao VA (2018). Specific protein carbonylation in human breast cancer tissue compared to adjacent healthy epithelial tissue. PloS one.

[17] Levine RL (1990). Determination of carbonyl content in oxidatively modified proteins. Methods in enzymology.

[18] Colombo, G. *et al*. Protein carbonylation in human bronchial epithelial cells exposed to cigarette smoke extract. *Cell Biology and Toxicology*, 10.1007/s10565-019-09460-0 (2019). 10.1007/s10565-019-09460-0 30648195

[19] Fedorova M, Bollineni RC, Hoffmann R (2014). Protein carbonylation as a major hallmark of oxidative damage: update of analytical strategies. Mass spectrometry reviews.

[20] Vemula V, Ni Z, Fedorova M (2015). Fluorescence labeling of carbonylated lipids and proteins in cells using coumarin-hydrazide. Redox Biology.

[21] Valeur, B. & Berberan-Santos, M. N. In Molecular Fluorescence 75–107 (2012).

[22] Dilek O, Sorrentino AM, Bane S (2016). Intramolecular Catalysis of Hydrazone Formation of Aryl-Aldehydes via ortho-Phosphate Proton Exchange. Synlett.

[23] Dilek Ö, Bane SL (2008). Synthesis of boron dipyrromethene fluorescent probes for bioorthogonal labeling. Tetrahedron Letters.

[24] Huang C (2011). beta1 integrin mediates an alternative survival pathway in breast cancer cells resistant to lapatinib. Breast cancer research: BCR.

[25] Nakaigawa N (2006). Inactivation of von Hippel-Lindau gene induces constitutive phosphorylation of MET protein in clear cell renal carcinoma. Cancer research.

[26] Pennacchietti S (2003). Hypoxia promotes invasive growth by transcriptional activation of the met protooncogene. Cancer cell.

[27] Granito A, Guidetti E, Gramantieri L (2015). c-MET receptor tyrosine kinase as a molecular target in advanced hepatocellular carcinoma. Journal of Hepatocellular Carcinoma.

[28] Bladier C, Wolvetang E, Hutchinson P, de Haan J, Kola I (1997). Response of a primary human fibroblast cell line to H2O2: senescence-like growth arrest or apoptosis?. Cell Growth Differ.

[29] Benfeitas, R., Uhlen, M., Nielsen, J. & Mardinoglu, A. New Challenges to Study Heterogeneity in Cancer Redox Metabolism. *Frontiers in Cell and Developmental Biology* **5**, 10.3389/fcell.2017.00065 (2017). 10.3389/fcell.2017.00065 PMC5504267 28744456

[30] Hudson CC (2002). Regulation of hypoxia-inducible factor 1alpha expression and function by the mammalian target of rapamycin. Molecular and cellular biology.

[31] Giandomenico AR, Cerniglia GE, Biaglow JE, Stevens CW, Koch CJ (1997). The importance of sodium pyruvate in assessing damage produced by hydrogen peroxide. Free Radic Biol Med.

[32] Jung T, Grune T (2013). The proteasome and the degradation of oxidized proteins: Part I-structure of proteasomes. Redox Biol.

[33] Davies KJ (2001). Degradation of oxidized proteins by the 20S proteasome. Biochimie.

[34] Tanase M (2016). Role of Carbonyl Modifications on Aging-Associated Protein Aggregation. Scientific Reports.

[35] Grune T, Jung T, Merker K, Davies KJ (2004). Decreased proteolysis caused by protein aggregates, inclusion bodies, plaques, lipofuscin, ceroid, and ‘aggresomes’ during oxidative stress, aging, and disease. The international journal of biochemistry & cell biology.

[36] Coliva, G., Duarte, S., Pérez-Sala, D. & Fedorova, M. Impact of inhibition of the autophagy-lysosomal pathway on biomolecules carbonylation and proteome regulation in rat cardiac cells. *Redox Biology*, 101123, 10.1016/j.redox.2019.101123 (2019). 10.1016/j.redox.2019.101123 PMC6859560 30737170

[37] Cannizzo ES, Clement CC, Sahu R, Follo C, Santambrogio L (2011). Oxidative stress, inflamm-aging and immunosenescence. Journal of proteomics.

[38] Salahudeen AK, Clark EC, Nath KA (1991). Hydrogen peroxide-induced renal injury. A protective role for pyruvate *in vitro* and *in vivo*. The Journal of clinical investigation.

[39] Srinivasan R, Ricketts CJ, Sourbier C, Linehan WM (2015). New Strategies in Renal Cell Carcinoma: Targeting the Genetic and Metabolic Basis of Disease. Clinical cancer research: an official journal of the American Association for. Cancer Research.

[40] Liemburg-Apers DC, Willems PHGM, Koopman WJH, Grefte S (2015). Interactions between mitochondrial reactive oxygen species and cellular glucose metabolism. Archives of Toxicology.

[41] Kuznetsov AV (2011). Mitochondrial ROS production under cellular stress: comparison of different detection methods. Analytical and bioanalytical chemistry.

[42] Goyeneche AA, Harmon JM, Telleria CM (2006). Cell death induced by serum deprivation in luteal cells involves the intrinsic pathway of apoptosis. Reproduction (Cambridge, England).

[43] Brodaczewska KK, Szczylik C, Fiedorowicz M, Porta C, Czarnecka AM (2016). Choosing the right cell line for renal cell cancer research. Molecular Cancer.

[44] Schmidt L (1999). Novel mutations of the MET proto-oncogene in papillary renal carcinomas. Oncogene.

[45] Chakraborty S (2019). Activation of c-Met in cancer cells mediates growth-promoting signals against oxidative stress through Nrf2-HO-1. Oncogenesis.

[46] Ozaki M, Haga S, Zhang HQ, Irani K, Suzuki S (2003). Inhibition of hypoxia/reoxygenation-induced oxidative stress in HGF-stimulated antiapoptotic signaling: role of PI3-K and Akt kinase upon rac1. Cell death and differentiation.

[47] Gloire G, Legrand-Poels S, Piette J (2006). NF-κB activation by reactive oxygen species: Fifteen years later. Biochemical Pharmacology.

[48] Portoghese PS, Sultana M, Takemori AE (1990). Design of peptidomimetic delta opioid receptor antagonists using the message-address concept. Journal of medicinal chemistry.

[49] Öztürk BÖ, Bucak E, Karabulut S (2013). *In situ* modification of the Grubbs first generation catalyst: A highly controllable metathesis catalyst bearing tridentate Schiff base ligands. Journal of Molecular Catalysis A: Chemical.

[50] Banerjee A (2010). Site-Specific Orthogonal Labeling of the Carboxy Terminus of α-Tubulin. ACS Chemical Biology.

[51] Lakowicz, J. R. *Principles of fluorescence spectroscopy*. (Second edition. New York: Kluwer Academic/Plenum, 1999).

[52] Nayman AH (2019). Dual-Inhibition of mTOR and Bcl-2 Enhances the Anti-tumor Effect of Everolimus against Renal Cell Carcinoma *In Vitro* and *In Vivo*. Journal of Cancer.

